# What Works in the Treatment of Borderline Personality Disorder

**DOI:** 10.1007/s40473-017-0103-z

**Published:** 2017-02-03

**Authors:** Lois W. Choi-Kain, Ellen F. Finch, Sara R. Masland, James A. Jenkins, Brandon T. Unruh

**Affiliations:** 1 Harvard Medical School, McLean Hospital, 115 Mill St., Mail Stop 312, Belmont, MA 02478 USA; 2 Massachusetts General Hospital, McLean Hospital, Belmont, MA 02478 USA

**Keywords:** Borderline personality disorder, Psychotherapy, General psychiatric management, Dialectical behavior therapy, Mentalization-based treatment, Transference-focused psychotherapy, Schema-focused therapy

## Abstract

**Purpose of the Review:**

This review summarizes advances in treatments for adults with borderline personality disorder (BPD) in the last 5 years.

**Recent Findings:**

Evidence-based advances in the treatment of BPD include a delineation of generalist models of care in contrast to specialist treatments, identification of essential effective elements of dialectical behavioral therapy (DBT), and the adaptation of DBT treatment to manage post-traumatic stress disorder (PTSD) and BPD. Studies on pharmacological interventions remain limited and have not provided evidence that any specific medications can provide stand-alone treatment.

**Summary:**

The research on treatment in BPD is leading to a distillation of intensive packages of treatment to be more broadly and practically implemented in most treatment environments through generalist care models and pared down forms of intensive treatments (e.g., informed case management plus DBT skills training groups). Evidence-based integrations of DBT and exposure therapy for PTSD provide support for changing practices to simultaneously treat PTSD and BPD.

## Introduction

Once thought to be an untreatable condition, borderline personality disorder (BPD) is now effectively treated by a growing number of evidence based psychotherapeutic treatments. Twenty-five years ago, Marsha Linehan published the first randomized control trial (RCT) for dialectical behavioral therapy (DBT), which yielded more significant reduction in the disorder’s most challenging features—parasuicidal behavior, inpatient psychiatric stays, and treatment drop out—than treatment as usual (TAU). Since then, over 13 manualized psychotherapies for BPD have been tested. Five major treatments—DBT, mentalization-based treatment (MBT) [1], schema-focused therapy (SFT) [2], transference-focused psychotherapy (TFP) [3], and systems training for emotional predictability and problem solving (STEPPS) [4]—have been established as evidence based treatments (EBTs) for BPD [5]. In contrast, trials testing psychopharmacologic treatments have not yielded consistent results [6, 7]; therefore, to date no medication has an official indication for BPD [8•]. This review will summarize major findings of what works in treatments for BPD, with a special emphasis on developments in the last 5 years.

The Cochrane review of psychological therapies for borderline personality disorder, which analyzed 28 studies published until 2011, is among the most significant additions to the literature on treatments for BPD in the last 5 years [5]. The major randomized controlled studies can be characterized in four major waves (Table 1). The first wave of studies compared specialized therapies for BPD to TAU. In this first wave of studies, DBT and MBT were established as EBTs [1, 9–11]. Additionally, a short-term group therapy, STEPPS, was added to TAU and found to be more effective than TAU alone in reducing symptoms of BPD, negative mood states, and impulsivity while increasing functioning [4].

**Table 1:** Major waves of RCTs for BPD treatment

Wave 1: vs. TAU		
DBT	Linehan et al. [11]	1991
DBT	Linehan et al. [10]	1994
DBT	Koons et al. [9]	2001
DBT	Carter et al. [12]	2010
DBT	Priebe et al. [13]	2012
DBT PTSD	Steil et al. [14]	2010
DBT PTSD	Bohus et al. [15•]	2013
MBT	Bateman & Fonagy [1]	1999
MBT-A	Rossouw & Fonagy [16]	2012
CBT+TAU	Davidson et al. [17]	2006
MACT	Weinberg et al. [18]	2006
STEPPS + TAU	Blum et al. [4]	2008
STEPPS + adjunctive therapy	Bos et al. [19]	2010
DDP	Gregory et al. [20]	2008
Wave 2: vs. community experts		
DBT	Linehan et al. [21]	2006
TFP	Doering et al. [22]	2010
Wave 3: vs manualized treatment		
SFT vs TFP	Giesen-Bloo et al. [2]	2006
DBT vs DDP vs TAU	Sachdeva et al. [23]	2013
TFP vs DBT vs supportive therapy	Clarkin et al. [3]	2007
Wave 4: vs. generalized treatment		
DBT vs GPM	McMain et al. [24]	2009
MBT vs SCM	Bateman & Fonagy [25]	2009
MBT vs supportive group therapy	Jørgensen et al. [26]	2013

Responding to criticisms that treatments lead by experts had an obvious advantage to TAU, investigators in subsequent trials in the second wave compared specialized BPD treatments, such as DBT and TFP, to treatment by expert therapists in the community, known for their willingness and interest in patients with BPD [21, 22]. DBT again showed higher reduction in suicidal behavior and self-injury, inpatient hospitalization, and treatment drop out than treatment by other experts in the community [21]. Similarly, in the trial comparing TFP to treatment by community experts, TFP yielded greater improvements in not only suicide attempts, inpatient hospitalization, and treatment drop out, but also in borderline symptoms, psychosocial functioning, and personality functioning [22]. Treatment by experts was as effective as specialty treatments in reducing depressive symptoms, and in the DBT trial, treatment by experts was effective in reducing suicidality, but not to the degree achieved in DBT [21, 22].

The third wave of studies staged head-to-head trials between specialist treatments (i.e., TFP vs. DBT [3]; SFT vs. TFP [2]). The evidence suggested a variety of systematic and well-informed manualized psychotherapies were effective at treating BPD and little was to be gained at horseracing to determine the superiority of any of them [27]. Clarkin’s TFP trial straddles both the third and fourth wave of studies [3]. It incorporated a systematic, manualized supportive therapy as a third comparison treatment to TFP and DBT. All three treatments, which were systematic and supervised, yielded improvements in depression, anxiety, and functioning [3]. While the supportive therapy arm failed to match reductions in suicidality yielded by both DBT and TFP, it proved effective enough to be an alternative to treating BPD patients in the absence of DBT and TFP treatments.

The other fourth wave of studies compared specialist therapies to systematic and well-informed generalist approaches to managing BPD. These studies aimed to show that the core ingredients to the specialist treatments provided increased gains in treatment, apart from the well organized and well-informed aspects of the clinical approach [3]. Unexpectedly, these enhanced, structured, and well-informed generalist treatment approaches performed as well in most ways to their already established specialized counterparts. Structured clinical management (SCM) [25, 28] and general psychiatric management (GPM) [8•, 24] emerged to provide less specialized means of managing BPD in the general patient population. Additionally, supportive therapy, when delivered in a systematic way [3] or by experienced clinicians in a group format [26], proved comparable in outcomes to TFP and MBT. When clinicians treating a general caseload of patients cannot often commit the time and expense to training for the gold standard specialist treatments for BPD [29], SCM, GPM, and supportive therapy offer practical, less intensive, “good enough” treatment options.

While generalist approaches to BPD could widen access to evidence-based care, research dismantling differing aspects of effective, but complex and intensive treatments, can clarify their essential elements. Linehan and colleagues published a dismantling study of DBT, illustrating that a simplified version of DBT, involving skills training group combined with weekly case management was effective in treating problems of self-harm, suicidality, and use of hospitalization [30•]. Generalist care, with the addition of targeted short-term adjunctive interventions in group formats or aimed at suicidality [4, 18, 31] may complement supportive or generalist approaches to yield good outcomes, with the investment of fewer clinical resources.

Investigators have also adapted the established evidence based treatments for BPD to manage the usual complex co-morbidities of BPD including substance use disorders substance use disorders (SUDs) [32], eating disorders (EDs) [33], and post-traumatic stress disorder (PTSD) [34]. BPD patients who present with acutely symptomatic co-morbidities of these types are often challenging to manage with strictly BPD oriented treatments [35]. Conversely, in SUD and ED treatments, individuals with co-morbid BPD may also present with problems that are difficult to manage in those treatment environments. Efforts to target BPD with its co-morbid disorders simultaneously have been developed and studies of their feasibility and effectiveness have been published in the last 5 years [15•, 32, 33, 36, 37, 38].

The remainder of this review will describe the prevailing evidence-based psychotherapies for BPD, the newer generalist management approaches for BPD, dismantling studies of evidence-based treatments (EBTs) for BPD, adaptations of EBTs for complex co-morbidities, and the current state of knowledge on psychopharmacologic interventions for BPD. This overview will demonstrate trends to paring down treatments to what essentially works for BPD, that most clinical settings can consider implementing as a more clear and feasible standard of care.

### Specialized Evidence-Based Treatments (EBTs) for BPD

#### Dialectical Behavioral Therapy (DBT)

The most well-known, well researched, and widely available EBT for BPD is DBT [39, 40]. Informed by clinical experience with suicidal personality disordered patients who did not improve with standard cognitive behavioral therapy intervention, Linehan developed DBT by incorporating the concept of dialectics and the strategy of validation into a treatment focused on skills acquisition and behavioral shaping. DBT formulates the problems of BPD as a result of the transaction between individuals born with high emotional sensitivity and “invalidating environments” that is, people or systems (i.e., families, schools, treatment settings, workplaces) that cannot perceive, understand, and respond effectively to their vulnerabilities.

DBT proposes that individuals with BPD can become more effective in managing their sensitivities and interactions with others through acquisition of skills that enhance mindfulness and enable them to better tolerate distress, regulate their emotions, and manage relationships. The full empirically validated package of DBT includes 1 h of weekly individual therapy, a 2-h group skills training session, out-of-session paging, and consultation team for the therapist. The intensity and structure of DBT, which is organized in an explicit, comprehensive set of manuals with instruction to therapists as well as hundreds of skills worksheets, provides an instant foundation for practitioners of any discipline or level of experience. DBT is designed for teams of clinicians and is among the most time intensive modalities for patients and clinicians. Its major mechanism of change occurs via acquisition and generalization of skills to be more emotionally regulated, mindful, and effective in the face of individual sensitivities.

#### Mentalization-Based Treatment (MBT)

Mentalization refers to the complex capacity human beings develop to imagine the thoughts and feelings in one’s own and other’s minds to understand interpersonal interactions [41]. Therein lies its mechanism of change. MBT proposes that BPD symptoms arise when a patient stops mentalizing, leading patients to operate from pathologically certainty about other’s motives, the disconnection from grounding influence of reality, and a desperate need for proof of feelings through action. Attachment interactions become hyperactivated, feeding into distress and difficulty coping, rather than providing safety and security, rendering the therapeutic process with BPD difficult.

MBT aims to stabilize the problems of BPD by strengthening the patient’s capacity to mentalize under the stress of attachment activation [41]. MBT therapists adopt a stance of curiosity, and “not knowing” in order to encourage patients to assess their emotional and interpersonal situation through a more grounded, flexible, and benevolent lens. Prioritizing the maintenance of mentalizing, MBT therapists support patients to think through hyperactivated states themselves, rather than providing prepackaged or intellectualized explanations, insights, or skills. Outpatient MBT involves 50 min of weekly individual therapy, 75 min of group therapy, and a reflecting team meeting which serves to support clinical team members in their mentalization in the process of treatment [25]. Developed within the National Health Services (NHS) in the United Kingdom, MBT provides a tenable model for treating personality disordered patients settings where patients and clinicians face scarce resources.

#### Transference-Focused Psychotherapy (TFP)

Based on the conceptualization of *borderline personality organization* introduced by Otto Kernberg in the 1960s, transference-focused psychotherapy (TFP) is a manualized, psychoanalytically oriented psychotherapy. Kernberg defined identity diffusion, primitive defense mechanisms (e.g., splitting), unstable reality testing, internally and externally expressed aggression, and conflicted internal working models of relationships as key features of personality disorders at borderline level of organization [42].

TFP’s focus is the problematic interpersonal dynamics in the patient’s life and their resultant intense emotional states. The patient’s inherent interpersonal dynamic emerge in interactions with the therapist in the transference, and are jointly examined to resolve the splits between good and bad that drive instabilities in affect and relationships. Like MBT, TFP’s mechanism of change occurs through helping patients achieve more balanced, integrated, and coherent ways of thinking about oneself and others. TFP involves two weekly individual therapy sessions, without group therapy. Clinicians in TFP are encouraged to receive supervision. TFP is more compatible with treatments by individual clinicians, not working in teams.

#### Schema-Focused Therapy (SFT)

Schema-focused therapy (SFT) is an integrative cognitive therapy focused on generating structural changes to a patient’s personality. In twice weekly individual therapy sessions, the clinician uses a variety of behavioral, cognitive, and experiential techniques that focus on the therapeutic relationship, daily life outside therapy, and past traumatic experiences. Unlike the more neutral stances of other therapies, SFT encourages an attachment between therapist and client, a process described as “limited re-parenting”. Therapy focuses on four schema modes of BPD: detached protector, punitive parent, abandoned/abused child, and angry/impulsive child. Its mechanism of change occurs through changing negative patterns of thinking, feeling, and behaving and developing healthier alternatives to replace them so that these dysfunctional schemas no longer control a patient’s life.

### Generalist Approaches to BPD

#### General Psychiatric Management (GPM)

Given the limitations of treatment models that require significant training and significant clinic resources, there is a need to develop, test, and disseminate less intensive treatments. One of these new EBTs is general psychiatric management (GPM) [8•].

GPM is based on a case management model, where interventions rely on common sense and are learned easily by generalist clinicians. Inherent in the case management approach is a focus on the patient’s life outside of therapy. GPM prioritizes the attainment of stable vocational functioning over romantic relationships, as well as improvement in social functioning over specific symptom improvement. Diagnostic disclosure with a discussion of the disorder’s symptoms should be the first step in psychoeducation for patients and their families, followed by information about the disorder’s etiology and positive prognosis. This frames a discussion of treatment frequency and duration—treatment is only provided if it is helping the patient progress based on articulated goals. GPM rarely involves more than one weekly individual appointment. The treatment is multimodal in nature and provides guidance for psychopharmacological interventions, as well as the provision of group and family therapy and coordination across providers. Its mechanism of change is to facilitate the natural course of the disorder’s improvement with specific attention to promoting functioning in endeavors outside of treatment.

What may be most specific to GPM is its central focus on an interpersonal hypersensitivity model of BPD [43]. In this model, the symptoms of BPD are understood as resulting from an emotional cascade that begins with an interpersonal stressor (e.g., separation, criticism). The therapist actively hypothesizes that any emotion dysregulation, impulsive or self-harming behavior, or hospitalization has resulted from an interpersonal problem, and works with the patient to better understand his or her sensitivities and responses.

In a randomized trial of GPM versus DBT, McMain et al. [24] found that patients in both treatments showed significant and comparable improvement across a variety of clinical outcomes measures after 1 year. Results were consistent at 2-year follow-up [44]. Furthermore, this trial later demonstrated that of patients with high Axis 1 co-morbidity, those assigned to GPM had significantly lower dropout rates than their DBT counterparts [45]. Moreover, there is evidence that attending a 1-day GPM workshop improves clinicians’ attitudes about treating BPD. For example, clinicians report feeling more hopeful, competent, and open to treat BPD [46]. These findings are particularly important given that BPD is a disorder for which significant stigma may introduce barriers to successful treatment. The success of treatment dissemination depends in large part on whether clinicians are willing to use treatments and feel competent to do so.

#### Structured Clinical Management (SCM)

Structured clinical management (SCM) was developed in the UK, similar to GPM, reflects “best general psychiatric treatment” that is feasible for use by “generalist mental health clinicians” with minimal additional training [47, p. 57]. It was developed based on “expert consensus” about what general practices work best for treating BPD, and has been tested primarily in the context of RCTs evaluating the effectiveness of MBT [25]. Compared to patients who received MBT, those who received SCM showed substantial improvements across an array of clinical outcomes. Patients receiving MBT improved somewhat more quickly and continued to show greater benefit than SCM at 18-month follow-ups. However, those who received SCM were as well at 6 months as those in the MBT group, and showed faster reductions in self-harm.

Like GPM, SCM provides a structured framework for approaching treatment for BPD (see Table 2 for comparisons). This framework is guided by a number of generalist principles and is meant to make treatment understandable and predictable for patients. There is an emphasis on sharing the borderline diagnosis with patients, psychoeducation, alliance building that is based both on contractual (e.g., goal agreement) and relational factors (e.g., trust, reliability, liking), encouragement of family involvement, limited reliance on psychopharmacological intervention, some guidance on managing co-morbid conditions, and explicit safety planning. Both GPM and SCM recommend intersession contact be used sparingly. However, SCM takes a more cautious approach, advocating for “vigorously supporting the patient on the telephone if necessary” [47, p. 69], vehemently pursuing clients who have not come to treatment, and a willingness to meet them at home or elsewhere when safety risk is elevated. This may have more to do with differences in the legal climate of the UK versus the USA than with beliefs about the utility of intersession contact. Also, SCM includes specifically articulated weekly group therapy. Group therapy is open on a rolling basis for patients and includes psychoeducation and a framework focused on problem solving.

**Table 2:** Comparison of GPM and SCM

	GPM [8]	SCM [47]
Focus	• Life outside treatment • Interpersonal hypersensitivity	• Symptom reduction • Social functioning
Clinician strategies	• Self-disclosure • Psychodynamic and behavioral principles • Flexibility/pragmatism • Relationship building • Case management • Psychoeducation	• Psychodynamic principles • Borrows from DBT, CBT, Rogerian therapy, and MBT • Case management • Psychoeducation
Individual therapy	• 1/week PRN if working	• 1/week
Group therapy	• Encouraged	• Required problem-solving group 1.5 h/wk
Family involvement	• Psychoeducation • Multi-family groups	• 2-session intervention • Further sessions PRN
Intersession contact	• Use sparingly	• Use sparingly • Case management PRN
Crisis management	• Safety planning • Chain analyses	• Support • Problem solving
Co-morbidities	• Specific hierarchies (e.g., BPD considered primary to MDD)	• BPD primary to MDD • Substance use/dependence managed with adjunctive care
Medications	• Conservative approach	• Conservative approach • Prescribe for co-morbid conditions not BPD

SCM has considerable similarity to GPM in terms of training requirements, structure, and general principles. However, descriptions of therapeutic techniques employed in SCM suggest that in some respects, it may appear more similar to MBT than GPM in practice. GPM is less psychotherapeutically oriented than other evidence-based treatments for BPD [29]. The general therapeutic stance includes responsivity, appropriate self-disclosure, flexibility, and pragmatism. The nonspecific techniques and therapeutic stance employed in SCM are generally rooted in psychodynamic principles consistent with MBT. These include authenticity and openness, the adoption of a “not knowing” stance, a focus on misunderstandings in the relationship, and generation of curiosity about belief and intentions.

### Dismantling Studies

Now that several evidence-based treatments for BPD have been tested, their most essential ingredients can be discerned from dismantling studies. A major advance in the last 5 years for understanding what works in BPD treatment comes from Linehan’s dismantling study of DBT. DBT in its standard form involves an intensive package of weekly individual therapy, weekly two and a half hour skills training group, 1-h consultation team for the therapist, and paging for skills coaching available between sessions. Linehan and colleagues compared this full, or “standard DBT” package to DBT individual therapy (DBT-I) without DBT skills training group as well as DBT skills training group (DBT-S) without skills coaching [30•]. All packages of DBT demonstrated significant improvements in suicidality and reduction in use of crisis services. While standard DBT showed greater improvement in frequency of self-harm, anxiety, and depression than the DBT-I condition, it did not show significant gains over DBT-S despite the significant difference in total hours of treatment (average 55.3 h in standard DBT versus 31.7 h in DBT-S). Linehan’s component analysis advances an imperative to distill the specialized and resource intensive EBTs for BPD to its most basic effective parts for the broadest, most resource efficient implementation.

### Other Brief Cost-Effective Options

#### Systems Training for Emotional Predictability and Problem Solving (STEPPS)

Designed to supplement ongoing treatments such as medication, individual therapy, and case management, systems training for emotional predictability and problem solving (STEPPS) consists of cognitive behavioral elements, skills training, and a systems component. The STEPPS program includes 20 weeks of 2-h seminar-like group sessions. Two co-facilitators lead the sessions by following detailed lesson plans that cover three main components of STEPPS. The first component, psychoeducation, focuses on reframing BPD as an “emotional intensity disorder,” correcting misconceptions about BPD, and helping participants gain an awareness of the schemas that drive their behaviors. The second, emotion management skills trainings, teaches skills to better manage the effects of BPD including distancing, communicating, and problem management. The third, behavior management skills training, covers goal setting, healthy eating, sleep, and exercise habits, self-harm avoidance, and interpersonal effectiveness. Participants are responsible for course materials and homework. The skills-based group sessions are supplemented by a single 2-h psychoeducation and skills training session for families which accounts for the systems component of STEPPS. While STEPPS is not designed to be a stand-alone treatment, a limited body of research indicates that STEPPS’ manualized, supplemental group sessions may enhance concurrent BPD treatments.

More recently, a series of studies conducted by Black and colleagues [48] explored the effectiveness of STEPPS for BPD patients with co-morbid antisocial personality disorder (ASPD) in both university and correctional settings. The findings indicate that subjects with co-morbid ASPD experienced similar or greater improvement in BPD symptoms, impulsiveness, and global symptoms than their non-ASPD counterparts and that STEPPS treatment is effective. These preliminary findings suggest that STEPPS has potential for treating a uniquely difficult to treat population: offenders with co-morbid BPD and ASPD.

### Treatments for BPD and Major Co-morbidities

BPD’s usual complex pattern of co-morbidity [49, 50] is another challenging factor in its clinical management. While depression is its most common co-morbidity, co-occurring with BPD in the majority of cases [50], evidence from RCTs presented here suggest it responds to specialist and generalist approaches, and tends to improve when BPD improves [51]. Other common co-morbidities such as substance use disorders (SUDS) [32], eating disorders (EDs) [33], and post-traumatic stress disorder (PTSD) [34] have been shown to lead to poorer treatment outcomes in some trials. While some evidence exists that these co-occurring disorders respond to DBT [35], MBT [52], and GPM [53], the combination of self-harm or suicidality with active substance dependence or eating disorder often makes these patients ineligible for specific treatment programs in clinical practice. The application and adaptation of some of the EBTs reviewed here have begun to elucidate direction in the simultaneous management of these challenging co-morbidities.

#### Substance Use Disorders (SUDs)

Two reviews of interventions for co-occurring BPD and SUDs were published in the last 5 years [32, 38]. These reviews indicate that DBT, in standard form as well as adapted in combination with opiate agonist medication, resulted in greater improvement of SUD, than either opiate agonist medication alone or treatment by community experts [35, 54]. In addition, dynamic deconstructive psychotherapy (DDP) [55], a manualized psychodynamic treatment for BPD and alcohol use disorders aimed at integrating and verbalizing emotional experiences as well as enhancing interpersonal identity and interactions through greater differentiation of self and other, showed greater improvements in BPD and alcohol use problems than TAU [20].

#### Eating Disorders

A limited number of studies have suggested that some individuals with co-morbid EDs and BPD will respond to standard DBT treatment [35, 56]. One study adapted DBT for EDs in a 3-month inpatient treatment, with improvement in both bulimia nervosa (BN) and anorexia nervosa (AN), resulting in remission rates higher than reported for standard DBT from other studies [33]. The impact of this modified form of DBT was more robust for BN than AN. MBT has been adapted to treat the combination of BPD and ED [37], but results of this trial are yet to be published.

#### Post-Traumatic Stress Disorder (PTSD)

Of the three complicating co-morbidities for BPD presented here, the clearest progress in treatment in the last 5 years has developed for treatment of BPD and PTSD. While BPD patients commonly have co-occurring PTSD (approximately 30% in community samples and 50% in clinical samples) [36], guidelines for treating both simultaneously did not exist until recently. Common practice prior to these studies assumed BPD would need attention first, and then PTSD work would follow. Standard PTSD protocols have been contraindicated in the context of active self-harm or suicidality [57]. Two different DBT research groups have published positive trials on adaptations of DBT for co-morbid PTSD, one adding exposure protocols to standard DBT [15•, 36] and the other modifying standard DBT to address severe PTSD symptoms at the outset of treatment [28]. The first of these trials [15•] focused on PTSD associated with the most complex and severe forms of trauma, for example childhood sexual abuse, in a 3-month residentially based treatment, reporting significant reductions in PTSD-related symptoms when compared to TAU (waitlist). This distilled adaptation to DBT focuses on exposure with anti-dissociation skills, emotional regulation regarding shame and guilt, and acceptance on the trauma and the feelings a patient has surrounding it. The second trial [36] compared standard DBT to DBT plus a DBT prolonged exposure protocol (DBT-PE), conducted in an outpatient setting over 12 months. This second smaller study demonstrated the superiority in the enhanced DBT-PE treatment in terms of reduction of PTSD-related symptom as well as suicidality and self-injury. These two trials suggest co-morbid PTSD and BPD can be concurrently treated with DBT and exposure protocols effectively. Bohus’s trial was a larger study involving 29 patients in a naturalistic treatment setting and a waitlist comparison group of 29 patients. It employed a pared down and concentrated version of DBT without a need to prioritize attention to self-harming behaviors, or to wait for a reduction in suicidality or self-harm to start the treatment. Harned’s trial was small, with ten of 17 DBT-PE patients completing the treatment, compared to five of nine completing standard DBT as its comparison group. The results of these trials suggest changing notions that these two disorders cannot be treated concurrently, or that significant stabilization need to occur prior to initiating PTSD focused work. Further research is needed to replicate these results.

### Pharmacology

Compared with the growing evidence base for effective psychological treatments, pharmacologic treatments for BPD remain less well-studied. To date, no medication has been approved by the FDA for BPD or proven to definitively manage its cardinal symptoms, interpersonal impairments, and functional difficulties. Clinical applicability of the available evidence is hampered by a limited number of studies, small sample sizes, non-overlapping batteries of outcome measures, brief observation periods, and exclusion of co-morbidities in a population for whom both psychiatric and medical co-morbidity are the rule rather than an exception.

Studies of prescribing practices indicate little conformity with the existing humble evidence-base. BPD patients are commonly subject to psychotropic polypharmacy, more likely to receive all classes of psychotropics, except TCAs and MAOIs [58]. After 16 years of prospective follow-up, 18.6 and 6.9% of BPD patients report taking four or more and five or more psychotropic medications, respectively [59]. In the same cohort over 14 years of follow-up, nearly one-third consistently used PRN medication despite the absence of any evidence-based protocols for prescribing PRNs for BPD [60]. The wish to be helpful and perceived “pressure to do something” for patients presenting in acute distress reporting unrelenting agitation, anxiety, and insomnia may account for the tendency to prescribe outside of evidence-based parameters.

In an attempt to offer sensible clinical roadmaps, practice guidelines have been proposed by regulatory organizations and thought leaders. The American Psychiatric Association (APA) guidelines proposed a symptom-targeted approach using specific medication classes to treat specific BPD symptom domains [61]. Their recommendations include the use of SSRIs for symptoms of affective dysregulation and impulsivity, and low-dose atypical antipsychotics for “cognitive-perceptual” symptoms such as transient stress-induced paranoia and dissociation. These guidelines have been criticized for being based on an artificial construct of the psychobiology of personality disorders, an overreliance on reconstructions of retrospective data, and the lack of adequately powered RCTs to inform their recommendations [62].

In contrast, the UK-based National Institute for Clinical Excellence (NICE) guidelines revised in 2015 conclude from its review of available RCTs that the evidence is not robust enough to inform any prescribing practices, citing the study limitations discussed above [63]. NICE recommends against using any medications for BPD or specific associated symptoms, but suggests they should be used for true co-morbidities and hesitantly allows their short-term use during acute crisis.

Subsequent meta-analyses have extended the “symptom domain” model that informed the APA guidelines to extrapolate across variable outcome measurements between individual studies. The Cochrane review meta-analysis found that although differences between drugs were not statistically different and effects were modest at best, improvement was seen in all symptom domains of BPD with psychopharmacological treatment [7]. Affective dysregulation was found to improve with haloperidol, aripiprazole, olanzapine, lamotrigine, divalproex, and topiramate, with no significant differences between these medications or classes. Behavioral dyscontrol/impulsivity improved with aripiprazole, topiramate, and lamotrigine. Cognitive-perceptual symptoms improved with aripiprazole and olanzapine. Interestingly, no SSRIs were found to improve any domain of symptoms and no medications alleviated certain core BPD symptoms, including avoidance of abandonment, chronic feelings of emptiness, identity disturbance, and dissociation. A second meta-analysis by Ingenhoven et al. identified a larger effect size for mood stabilizers on the treatment of behavioral dyscontrol-impulsive behaviors and anger and a more pronounced effect on global functioning, especially for lamotrigine and topiramate [64].

Of the psychotherapeutic approaches reviewed here, only GPM and SCM incorporates specific psychopharmacological guidelines, which combine elements of NICE and APA guidelines [8•]. Principled, yet flexible approaches to prescribing attempts to strike a balance between the evidence-based perspective that medications appear at best adjunctive while also being potentially harmful and the clinical wisdom that medication may be cautiously used in a symptom-targeted manner to promote engagement in psychotherapy. Prescribers are encouraged to avoid reactive prescribing and deliver psychoeducation about suggested medications which includes appropriate uncertainty about expected benefits.

GPM offers a specific algorithm to help prescribers limit harm and maximize potential benefit from medications. In this algorithm, no medications are prescribed for patients who do not request them and are not in distress. SSRIs are prescribed for either bona fide co-morbid major depressive episode or for patients who may have mild distress and request a medication. For behavioral dyscontrol/impulsivity and anger, either a mood stabilizer or antipsychotic can be prescribed, but risks must be discussed. For those with cognitive-perceptual disturbance, low-dose antipsychotics are recommended. In keeping with NICE guidelines, GPM promotes discontinuing medications if ineffective, avoiding polypharmacy, and time-limiting use.

Existing guidelines for pharmacotherapy in BPD do not reflect consensus but rather a chorus of distinct voices with a few overlapping refrains. One shared aim is the minimization of harms by prioritizing psychotherapy whenever possible, avoiding polypharmacy, adding medications and tapering those lacking clear efficacy in a stepwise fashion, promoting patients’ collaboration through psychoeducation and joint monitoring of risks and benefits, and considering the usefulness of prescribing in view of effects on the therapeutic relationship. Finally, benzodiazepines and benzodiazepine-like substances should be avoided out of concern for promoting dependence, abuse, and behavioral disinhibition.

## Conclusion

Prior to 2011, a number of highly specialized psychotherapies, (e.g., DBT, MBT, TFP, and SFT) entered the clinical scene as effective EBTs for BPD. The proliferation of these intensive EBTs turned the tide of prevailing notions that BPD was an untreatable condition. However, while effective treatments became more available, the intensity of EBTs for BPD suggested that BPD could best be treated by specialists. More recently, structured generalist management approaches such as GPM and SCM, have also been proven to work for patients with BPD. A dismantling study of DBT has offered evidence that a more pared down version of DBT, with skills training group and case management is nearly as effective as standard resource intensive packages of DBT.

Now less intensive forms of effective treatment for BPD are more available, providing hope that the general standard of care for these complex patients can be improved. Some proposals for determining which patients should received generalist care versus specialized care have recommended that patients with either greater personality dysfunction (i.e., greater numbers of personality disorder diagnoses) [52] or lack of response to generalist models of care [29] be allocated to a higher more intensive EBT. In contrast to psychotherapy trials, pharmacology trials suggest medications are adjunctive at best, and best minimized except in the treatment of co-morbidities.

Lastly, the BPD treatment research has paid increasing attention to co-morbidities such as SUDs, EDs, and PTSD. While evidence is accumulating for treatment studies in this area, clear advances in adapting DBT to treatment for PTSD has been made in the last 5 years. Now, exposure protocols for treating PTSD have been incorporated into DBT to help patients with both BPD and PTSD symptoms. Further research on adaptations of treatment for patients with more complex co-morbidities is still needed.
